# Relationship between body composition indicators and risk of type 2 diabetes mellitus in Chinese adults

**DOI:** 10.1186/s12889-020-08552-5

**Published:** 2020-04-06

**Authors:** Yongchun Chen, Dongliang He, Tingting Yang, Hao Zhou, Siyun Xiang, Lijun Shen, Jing Wen, Shengcai Chen, Songxu Peng, Yong Gan

**Affiliations:** 1 Department of Nutrition, Henan Provincial People’s Hospital and Zhengzhou University People’s Hospital, Zhengzhou, Henan 450003 People’s Republic of China; 2 Department of Nutrition, Hengyang Central Hospital, Hengyang, Hunan 421001 People’s Republic of China; 3 Department of Medical Record, Fuwai Central China Cardiovascular Hospital, Zhengzhou, Henan 451464 People’s Republic of China; 4 Department of Research and Discipline Development, Henan Provincial People’s Hospital and Zhengzhou University People’s Hospital, Zhengzhou, 450003 People’s Republic of China; 5 Department of Neurology, Union Hospital, Tongji Medical College, Huazhong University of Science and Technology, No. 1277 Jiefang Avenue, Wuhan, 430022 Hubei People’s Republic of China; 6 Department of Maternal and Child Health, Xiangya School of Public Health, Central South University, No. 110, Xiangya Road, Changsha, 410008 People’s Republic of China; 7 Department of Social Medicine and Health Management, School of Public Health, Tongji Medical College, Huazhong University of Science and Technology, No. 13 Hangkong Road, Wuhan, Hubei 430030 People’s Republic of China

**Keywords:** Body composition, Type 2 diabetes mellitus, Predictive value, Indicators, Visceral fat area

## Abstract

**Background:**

Body composition is a crucial factor associated with the incidence of type 2 diabetes mellitus. However, no study on this relationship has been performed in the Chinese population. This study aimed to investigate the relationship between body composition indicators and risk of type 2 diabetes mellitus among Chinese adults undergoing medical examination.

**Methods:**

Between January 2018 and July 2018, a retrospective cross-sectional study was performed on 3367 (2307 male and 1060 female) participants aged ≥18 years undergoing medical examination in Zhengzhou. Logistic regression analysis was performed to explore the relationship between body composition indicators and risk of type 2 diabetes mellitus. A receiver operating characteristic curve was used to calculate cutoff points and the predictive power of each indicator.

**Results:**

Among the 3367 participants, 12.53% were diagnosed with type 2 diabetes mellitus. Multivariate logistic analysis indicated that male participants (odds ratio [OR] = 1.68, 95% confidence interval [CI]: 1.29–2.19), older participants (OR = 1.05, 95% CI: 1.04–1.06), participants with a waist-to-hip ratio above the reference value (OR = 1.56, 95% CI: 1.18–2.07), participants with body fat percentage above the reference value (OR = 1.62, 95% CI: 1.01–2.68), and participant with a large visceral fat area (OR = 1.01, 95% CI: 1.01–1.02) had a high risk of type 2 diabetes mellitus. Waist-to-hip ratio, body fat percentage, and visceral fat area were the best indicators of type 2 diabetes mellitus (*P* < 0.001) with cutoff values of 0.90, 25.02%, and 92.00 cm^2^, respectively.

**Conclusion:**

This study suggests a predictive relationship between type 2 diabetes mellitus and body composition indicators of waist-to-hip ratio, body fat percentage, and visceral fat area, which are valuable for screening diabetes and providing effective health education and behavioral intervention for high-risk populations.

## Background

Diabetes is a condition that results from defective regulation of blood glucose, resulting in increased blood glucose levels [1–3]. Type 2 diabetes mellitus (T2DM) is the most common form of diabetes, accounting for approximately 90% of diabetes cases. The 2017 International Diabetes Federation (IDF) report showed that the number of diabetes patients in China reached 114.4 million in 2017, with 61.3 million (53.6%) newly diagnosed patients [4].

The increased incidence of T2DM has been linked to several risk factors, including population aging, economic development, urbanization, and obesity due to sedentary lifestyles and excessive eating [5–7]. Excessive accumulation of visceral fat leads to an imbalance in endocrine function and the release of pro-inflammatory factors, which may result in the development of insulin resistance and T2DM [8, 9]. Therefore, it is important to explore an effective measurement method to assess obesity—especially abdominal obesity—for early diagnosis and treatment of diabetes. Previous studies have explored body mass index (BMI), waist circumference, waist-to-hip ratio, and waist-to-height ratio as measures of obesity [10–12]. However, these measures are limited in their assessment of body composition and lack sensitivity in diagnosing T2DM. Recently, bioelectrical impedance analysis (BIA) has been developed to measure body composition [13], which has been shown to be a more convenient and less invasive method for assessing abdominal obesity and fat distribution.

Although there have been various epidemiological studies to determine the association between body composition indicators and risk of T2DM in other countries [14–16], no such study has been performed in China, the most populated country in the world.

This study aimed to investigate the relationship between body composition indicators and the risk of T2DM in Chinese adults undergoing medical examination. In addition, the receiver operating characteristic (ROC) curve was used to determine the best cutoff value of body composition indicators. These findings will not only provide a reference for T2DM screening and primary means for prevention but also enrich the research pool within the field of diabetes.

## Material

### Study population

The data of 3367 (2307 male and 1060 female) participants who underwent medical screening conducted between January 2018 and July 2018 at a tertiary hospital in Henan Province, China, were retrospectively analyzed. The participants were included if they met the following criteria: (1) aged ≥18 years and (2) examination of body composition using the BIA. The following participants were excluded from the study: those with (1) a history of chronic liver and kidney disease, thyroid disease, parathyroid disease, cancers, and type 1 diabetes; (2) a history of taking steroids; and (3) a history of taking hormonal agents. The inclusion/exclusion information was obtained from medical records and pre-examination inquiries.

### Measurement methods

#### Blood glucose

A total of 2 mL of venous blood was collected in the fasted state. Blood glucose level was then measured with a glucometer. According to the IDF Diabetes Atlas (8th Edition, 2017), the diagnostic criteria for diabetes were as follows: (1) fasting blood glucose ≥7.0 mmol/L (126 mg/dL), (2) blood glucose 2 h after the 75-g oral glucose tolerance test ≥11.1 mmol/L (200 mg/dL), (3) random blood glucose > 11.1 mmol/L (200 mg/dL), and (4) fulfillment of any one of the three criteria mentioned above or prior history of diabetes [4].

#### Body composition analysis

Body composition was measured by BIA using the InBody 770 (InBody Co., Ltd., Seoul, Korea), which consists of a patented 8-point tactile electrode system that estimates segmental composition, including body water, muscle mass, waist-to-hip ratio, body fat percentage, and visceral fat area (VFA). This widely used measurement is safe, non-invasive, and highly precise with comprehensive indicators and short measurement time [17]. Before the examination, the participants were required to empty their bowels and bladder, maintain a fasted state, wear light clothes, expose their limbs for electrode attachment, and rest for 5 min to ensure that accurate measurements were captured.

#### Judgment standards

The participants were deemed to have T2DM if they had a fasting blood glucose level of ≥7.0 mmol/L (126 mg/dL), if they had a 2 h postprandial blood glucose level of > 11.1 mmol/L (200 mg/dL), or in cases of self-reported diabetes [18]. Waist-to-hip ratio was classified as normal or over-standard with cutoff levels for normal classification at 0.9 for males and 0.8 for females [19]. The normal range of body fat percentages in males and females was 10–20% and 18–28%, respectively. On the basis of the criteria for overweight and obesity reported by the Working Group on Obesity in China, a BMI of ≥24.0 kg/m^2^ was considered to indicate overweight or obesity and a BMI of < 24.0 kg/m^2^ was considered normal or low [20]. All the participants were classified into normal/low or over-standard groups.

### Statistical analysis

Mean ± standard deviation was used for continuous variables, while counts and percentages were used for categorical data. Student’s *t*-test and the Chi-square test were applied to examine the differences in T2DM and its subscales among groups. Multiple logistic regression analysis was performed to estimate the predictors of T2DM. The area under the ROC curve was used to predict the optimal cutoff point for T2DM. Statistical analysis was performed using IBM statistics (SPSS) V.17. The significance level was set at α = 0.05.

## Results

Overall, 3367 participants were included in the study: 2307 (68.52%) males and 1060 (31.48%) females. The average age of the participants was 52.57 ± 15.61 years. In total, 1974 participants (58.63%) were deemed overweight or obese (BMI ≥ 24.0 kg/m^2^). In the over-standard group, there were 2477 (73.57%) and 2909 (86.40%) participants, on the basis of their waist-to-hip ratio and body fat percentage, respectively. The average VFA of the participants was 94.76 ± 34.01 cm^2^. Among the participants, 12.53% were diagnosed with T2DM (Table 1).

**Table 1:** General situation analysis of medical examination population

Characteristics	Number (n)	Percentage (%)
Sex		
Males	2307	68.52
Females	1060	31.48
Age($\overline{x}$*± s*, years)	52.57 ± 15.61	
BMI		
< 24.0 kg/m^2^	1393	41.37
≥ 24.0 kg/m^2^	1974	58.63
Waist-to-hip ratio		
Normal	890	26.43
Over-standard	2477	73.57
Body fat percentage		
Normal or under	458	13.60
Over-standard	2909	86.40
VFA($\overline{x}$*± s*, cm^2^)	94.76 ± 34.01	
Type 2 diabetes		
Yes	422	12.53
No	2945	87.47

*Abbreviations*: *BMI* body mass index, *VFA* visceral fat area

Differences in the indicators among participants with and without T2DM were analyzed by the chi-square test and independent sample *t*-test. The results showed that the detection rate of T2DM was higher for males than for females (14.35% vs. 8.58%, *χ*^2^ = 22.00, *P* < 0.001). The mean age was higher in theT2DM group than in the no-T2DM group (63.02 ± 13.21 years vs. 51.07 ± 15.35 years, t = 14.76, *P* < 0.001). The frequency of waist-to-hip ratio above the reference value (13.85%), obesity or overweight by BMI (14.74%), body fat percentage above the reference value (13.78%), and mean VFA (104.60 ± 34.71 cm^2^) was higher in the T2DM group than in the no-T2DM group. The differences were all statistically significant (*P* < 0.05) (Table 2).

**Table 2:** Comparison of relevant indicators between participants with and without T2DM

Variables	With T2DM	Without T2DM	***χ***^**2**^/***t***	***P***
Sex (n, %)			22.00	< 0.001
Males	331 (14.35)	1976 (85.65)		
Females	91 (8.58)	969 (91.42)		
Age($\overline{x}$*± s*, years)	63.02 ± 13.21	51.07 ± 15.35	17.02	< 0.001
Waist-to-hip ratio (n, %)			14.76	< 0.001
Normal	79 (8.88)	811 (91.12)		
Over-standard	343 (13.85)	2134 (86.15)		
BMI (n, %)			21.22	< 0.001
< 24.0 kg/m^2^	131 (9.40)	1262 (90.60)		
≥ 24.0 kg/m^2^	291 (14.74)	1683 (85.26)		
Body fat percentage (n, %)			30.55	< 0.001
Normal or under	21 (4.59)	437 (95.41)		
Over-standard	401 (13.78)	2508 (86.22)		
VFA($\overline{x}$*± s,* cm^2^)	104.60 ± 34.71	93.34 ± 33.67	6.40	< 0.001

*Abbreviations*: *BMI* body mass index, *VFA* visceral fat area

After adjusting for age and sex, the results of logistic regression analysis for risk factors of T2DM showed that the over-standard waist-to-hip ratio (odds ratio [OR] = 1.56, 95% confidence interval [CI]: 1.18–2.07, *P* = 0.002), body fat percentage above the reference value (OR = 1.62, 95% CI: 1.01–2.68, *P* = 0.049), and large VFA (OR = 1.01, 95% CI: 1.01–1.02, *P* = 0.001) are important predictors of T2DM (Table 3).

**Table 3:** Logistic regression analysis of risk factors of T2DM in medical examination population

Characteristics	***β***	***SE***	***Wald***	***OR***(95%***CI***)^a^	***P-value***
Constant term	− 7.82	0.59	177.87		< 0.001
Sex (Females as a reference)					
Males	0.52	0.14	14.84	1.68 (1.29–2.19)	< 0.001
Age (years)	0.05	0.00	168.55	1.05 (1.04–1.06)	< 0.001
Waist-to-hip ratio (normal as a reference)					
Over-standard	0.45	0.14	9.84	1.56 (1.18–2.07)	0.002
Body fat percentage (normal or low as a reference)					
Over-standard	0.48	0.26	3.60	1.62 (1.01–2.68)	0.049
VFA (cm^2^)	0.01	0.00	11.23	1.01 (1.01–1.02)	0.001

*Abbreviations*: *OR* odds ratio, *VFA* visceral fat area, *CI* confidence interval, *T2DM* type 2 diabetes mellitus

^a^*OR* adjusted for BMI

The ROC curves of the significant body composition indicators (waist-to-hip ratio, body fat percentage, VFA) from regression analysis were used to further determine the predictive effect of those indicators on T2DM. According to the ROC curves, the waist-to-hip ratio, body fat percentage, and VFA had predictive effects on T2DM (*P* < 0.001) among all participants with predictive cutoff values of 0.90, 25.02%, and 92.00 cm^2^, respectively. Among male participants, the predictive effects of waist-to-hip ratio, body fat percentage, and VFA on T2DM were statistically significant (*P* < 0.05) with predictive cutoff values 0.93, 25.02%, and 74.15 cm^2^, respectively. Among female participants, the predictive effects of waist-to-hip ratio, body fat percentage, and VFA on T2DM were statistically significant (*P* < 0.05) with predictive cutoff values of 0.89, 36.60%, and 90.15 cm^2^, respectively (Table 4).

**Table 4:** Analysis of the predictive effect of body composition indicators on T2DM

Body composition index	Classification	AUC	***P***	AUC95%***CI***	Cutoff points	Sensitivity	Specificity	Youden index
Waist-to-hip ratio	All	0.60 ± 0.02	< 0.001	0.58–0.63	0.90	0.706	0.458	0.164
	Males	0.58 ± 0.02	< 0.001	0.54–0.61	0.93	0.544	0.598	0.142
	Females	0.63 ± 0.03	< 0.001	0.58–0.69	0.89	0.582	0.628	0.210
Body fat percentage(%)	All	0.55 ± 0.01	0.001	0.52–0.58	25.02	0.810	0.273	0.083
	Males	0.58 ± 0.02	< 0.001	0.54–0.61	25.02	0.767	0.359	0.126
	Females	0.66 ± 0.03	< 0.001	0.60–0.71	36.60	0.516	0.739	0.255
VFA (cm^2^)	All	0.60 ± 0.01	< 0.001	0.57–0.62	92.00	0.600	0.540	0.140
	Males	0.58 ± 0.02	< 0.001	0.55–0.61	74.15	0.819	0.303	0.122
	Females	0.67 ± 0.03	< 0.001	0.62–0.73	90.15	0.813	0.464	0.277

*Abbreviations*: *AUC* area under curve, *CI* confidence interval, *SD* standard deviation, *VFA* visceral fat area, *T2DM* type 2 diabetes mellitus

## Discussion

Diabetes is the third leading cause of death worldwide, posing a serious threat to human health and exerting a heavy burden on patients, patients’ families, and society [21–23]. Epidemiological studies have shown that effective prevention and control of T2DM can have enormous social and economic benefits by delaying or reducing the onset of disease and related complications [24]. The participants in this study were a group of people undergoing medical examination in a Grade A Class 3 hospital in Henan Province. The prevalence of T2DM was 12.53% in this study, which was higher than the prevalence reported by Yang W et al. in 2007, i.e., 9.7% [25] and closer to the prevalence reported by Xu Y et al. in 2010, i.e., 11.6% [26]. This difference may be due to the different screening conditions of participants.

T2DM is known to have an insidious onset and can easily lead to serious complications that threaten human health. Accelerated rhythm of life, unhealthy diet, and sedentary lifestyle lead to storage of excess nutrients in the body and changes in body composition, resulting in metabolic disturbances [27]. Studying the relationship between body composition and T2DM is thus beneficial, as measurements of body composition can enable timely and effective identification of risk factors for T2DM, facilitating its early prevention.

Logistic regression analysis showed that male sex, old age, waist-to-hip ratio over the reference value, body fat percentage over the reference value, and large VFA were positively correlated with T2DM. The prevalence rate of T2DM was 1.68 times higher in males than in females, likely due to differences in lifestyle and work stress [25, 26]. In addition, the risk of T2DM increased because of changes in metabolism related to old age. Old age has become one of the established risk factors for T2DM. The results showed that while waist-to-hip ratio, body fat percentage, and VFA were associated with T2DM, BMI was not significantly associated with it. This suggested that the accumulation of abdominal fat is positively correlated with the onset of T2DM, and future research must explore the relationship between abdominal obesity and T2DM on a larger scale.

The ROC curve was used to evaluate the predictive value of various body composition indicators associated with the risk of T2DM. The average age of the study population was 52.57 ± 15.61 years. Because of differences in physiological changes and metabolism between males and females, in this study, we conducted a sex-based analysis apart from the overall population study to clearly show the risk factors of T2DM across sexes. Among all participants, the waist-to-hip ratio had a stronger predictive effect on T2DM with an optimal cutoff value of 0.90. Among male participants, the waist-to-hip ratio and body fat percentage showed strong predictive effects with optimal cutoff values of 0.93 and 25.02%, respectively. The predictive effect of the waist-to-hip ratio on T2DM in the male population suggests an adverse risk of abdominal obesity in males. Reducing abdominal fat accumulation may reduce the occurrence and development of male T2DM.

Among female participants, VFA had the strongest predictive effect on T2DM with a cutoff point of 90.15 cm^2^, which was lower than the previous VFA cutoff value i.e. 100 cm^2^ [28]. The lower predictive cutoff value may be attributed to population diversity. Among all body composition indicators, body fat percentage and VFA showed the strongest predictive effects on T2DM. Monitoring body composition indicators, specifically body fat percentage and VFA, can help identify underlying obesity in a timely manner, which is of great significance for reducing the onset and complications of T2DM.

This study has some limitations. First, this study was a cross-sectional study design, which restricted the predictive effect of the temporality and causality of the observed relationships. Second, the participants were selected from the medical examination population in one hospital, thus making selection bias unavoidable. Third, other potential predictors of T2DM (socioeconomic status, history of chronic disease, etc.) were not studied in our research. In the future, multi-center cohort studies, covering populations from different regions and including more influential factors, should be considered to verify the results of this study.

## Conclusions

The results of this study showed a high prevalence rate of T2DM associated with body composition indicators. Age, sex, waist-to-hip ratio, body fat percentage, and VFA were independent risk factors for T2DM. Waist-to-hip ratio and VFA had strong predictive effects on T2DM. The comprehensive measurement of human body composition and associated risk assessment system could provide an important diagnostic tool for individualized prevention of T2DM.

## Data Availability

Data may be made available by contacting the corresponding author on reasonable request with permission from Y.G..

## Abbreviations

AUC: Area under curve
BMI: Body mass index
CI: Confidence interval
HbA1c: Hemoglobin A1c
OR: Odds ratio
ROC: Receiver operating characteristic
SD: Standard deviation
VFA: Visceral fat area
T2DM: Type 2 diabetes mellitus

## References

[1] Evans JM, Newton RW, Ruta DA (2000). Socio-economic status, obesity and prevalence of type 1 and type 2 diabetes mellitus. Diabet Med.

[2] Bruno G, Runzo C, Cavallo-Perin P (2005). Incidence of type 1 and type 2 diabetes in adults aged 30-49 years: the population-based registry in the province of Turin, Italy. Diabetes Care.

[3] Holman N, Young B, Gadsby R (2015). Current prevalence of type 1 and type 2 diabetes in adults and children in the UK. Diabet Med.

[4] International Diabetes Federation. IDF Diabetes Atlas. Eight edition. 2017. https://www.idf.org/e-library/epidemiology-research/diabetes-atlas/134-idf-diabetes-atlas-8th-edition.html.

[5] Mozaffarian D (2016). Dietary and policy priorities for cardiovascular disease, diabetes, and obesity: a comprehensive review. Circulation..

[6] Forouhi NG, Wareham NJ (2014). The EPIC-InterAct study: a study of the interplay between genetic and lifestyle behavioral factors on the risk of type 2 diabetes in European populations. Curr Nutr Rep.

[7] Ley SH, Hamdy O, Mohan V (2014). Prevention and management of type 2 diabetes: dietary components and nutritional strategies. Lancet..

[8] Lv X, Zhou W, Sun J, Lin R, Ding L, Xu M (2017). Visceral adiposity is significantly associated with type 2 diabetes in middle-aged and elderly Chinese women: a cross-sectional study. J Diabetes.

[9] Bays HE (2011). Adiposopathy is “sick fat” a cardiovascular disease?. J Am Coll Cardiol.

[10] Bertrand LA, Thomas LJ, Li P (2017). Obesity as defined by waist circumference but not body mass index is associated with higher renal mass complexity. Urol Oncol.

[11] Borel AL, Coumes S, Reche F (2018). Waist, neck circumferences, waist-to-hip ratio: which is the best cardiometabolic risk marker in women with severe obesity? The SOON cohort. PLoS One.

[12] Liu L, Wang Y, Zhang W (2019). Waist height ratio predicts chronic kidney disease: a systematic review and meta-analysis, 1998-2019. Arch Public Health.

[13] Dulloo AG, Jacquet J, Solinas G (2010). Body composition phenotypes in pathways to obesity and the metabolic syndrome. Int J Obes.

[14] Kim EH, Kim HK, Bae SJ (2018). Gender differences of visceral fat area for predicting incident type 2 diabetes in Koreans. Diabetes Res Clin Pract.

[15] Tuzun S, Cifcili S, Dabak MR (2018). Sarcopenia among genders in type 2 diabetes mellitus patients using different formulas of bioimpedance analysis. J Coll Physicians Surg Pak.

[16] Solanki JD, Makwana AH, Mehta HB (2015). Body composition in type 2 diabetes: change in quality and not just quantity that matters. Int J Prev Med.

[17] Zhao ML (2016). The principle and the use of body composition measurement based on bioelectrical impedance analysis. Parenter Enteral Nutr.

[18] American Diabetes Association (2017). Classification and diagnosis of diabetes. Diabetes Care.

[19] Zhong JY, Wan Q (2018). Correlation between waist-to-hip ratio with hyperglycemia, hyperlipidemia and hyperuricemia in physical examination population. Lab Med Clin.

[20] China overweight/obesity medicine nutrition treatment expert consensus writing committee (2016). China overweight/obesity medical nutrition treatment experts consensus (2016). Diabetes World.

[21] Zimmet P, Alberti KG, Shaw J (2001). Global and societal implications of the diabetes epidemic. Nature..

[22] Stumvoll M, Goldstein BJ, van Haeften TW (2005). Type 2 diabetes: principles of pathogenesis and therapy. Lancet..

[23] Vos T, Barber RM, Bell B (2015). Global, regional, and national incidence, prevalence, and years lived with disability for 301 acute and chronic diseases and injuries in 188 countries, 1990-2013: a systematic analysis for the Global Burden of Disease Study 2013. Lancet..

[24] Ali MK, Bullard KM, Saaddine JB (2013). Achievement of goals in U.S. diabetes care, 1999-2010. N Engl J Med.

[25] Yang W, Lu J, Weng J (2010). Prevalence of diabetes among men and women in China. N Engl J Med.

[26] Xu Y, Wang L, He J (2013). Prevalence and control of diabetes in Chinese adults. JAMA..

[27] Weinstein AR, Sesso HD, Lee IM (2004). Relationship of physical activity vs body mass index with type 2 diabetes in women. JAMA..

[28] Li X, Katashima M, Yasumasu T (2012). Visceral fat area, waist circumference and metabolic risk factors in abdominally obese Chinese adults. Biomed Environ Sci.

